# QTL mapping for bioenergy traits in sweet sorghum recombinant inbred lines

**DOI:** 10.1093/g3journal/jkab314

**Published:** 2021-09-14

**Authors:** Vander Fillipe de Souza, Guilherme da Silva Pereira, Maria Marta Pastina, Rafael Augusto da Costa Parrella, Maria Lúcia Ferreira Simeone, Beatriz de Almeida Barros, Roberto Willians Noda, Luciano da Costa e Silva, Jurandir Vieira de Magalhães, Robert Eugene Schaffert, Antonio Augusto Franco Garcia, Cynthia Maria Borges Damasceno

**Affiliations:** 1 Embrapa Maize and Sorghum, Sete Lagoas, MG, 35701-970, Brazil; 2 Department of Genetics, Luiz de Queiroz College of Agriculture, University of São Paulo, Piracicaba, SP, 13418-900, Brazil; 3 JMP Division, SAS Institute Inc., Cary, NC 27513, USA

**Keywords:** *Sorghum bicolor*, bioethanol, quantitative trait loci, RIL, genotyping by sequencing

## Abstract

During the past decade, sweet sorghum (*Sorghum bicolor* Moench L.) has shown great potential for bioenergy production, especially biofuels. In this study, 223 recombinant inbred lines (RILs) derived from a cross between two sweet sorghum lines (Brandes × Wray) were evaluated in three trials. Single-nucleotide polymorphisms (SNPs) derived from genotyping by sequencing of 272 RILs were used to build a high-density genetic map comprising 3,767 SNPs spanning 1,368.83 cM. Multitrait multiple interval mapping (MT-MIM) was carried out to map quantitative trait loci (QTL) for eight bioenergy traits. A total of 33 QTLs were identified for flowering time, plant height, total soluble solids and sucrose (five QTLs each), fibers (four QTLs), and fresh biomass yield, juice extraction yield, and reducing sugars (three QTLs each). QTL hotspots were found on chromosomes 1, 3, 6, 9, and 10, in addition to other QTLs detected on chromosomes 4 and 8. We observed that 14 out of the 33 mapped QTLs were found in all three trials. Upon further development and validation in other crosses, the results provided by the present study have a great potential to be used in marker-assisted selection in sorghum breeding programs for biofuel production.

## Introduction

Sorghum (*Sorghum bicolor* Moench L.) is a diverse crop, which has contributed to the development of cultivars for different purposes and products, such as fodder, grains, and biofuels. Sweet sorghum cultivars have succulent stalks rich in sugars, similar to its close relative, sugarcane (*Saccharum* spp.). Moreover, sorghum is a versatile crop adapted to diverse soil and climate conditions, making it a promising alternative for energy production around the world (Ahmad Dar *et al.* 2018). In Brazil, the use of sweet sorghum as a complementary bioenergy feedstock could increase ethanol production efficiency not only by reducing the idle capacity of sugarcane mills during their off-season but also by increasing bioelectricity production from residual biomass (Jonker *et al.* 2015). The most important target traits in sorghum breeding programs focusing on biofuel production include shortening crop cycle duration, and increasing plant height, total biomass yield, and sugar or fiber contents (Regassa and Wortmann 2014). Such traits are quantitative in nature, controlled by many quantitative trait loci (QTL) (Mace *et al.* 2019), most of which might show a varied degree of genotype-by-environment (G × E) interactions.

Several QTL mapping studies for bioenergy traits involving sweet sorghum have already been reported (see Mathur *et al.*, 2017). However, these previous studies have generally relied on recombinant inbred line (RIL) populations generated from crosses between sweet and nonsweet sorghum lines genotyped with a rather low number of markers. Modern genotyping technologies based on next-generation sequencing (NGS), such as genotyping by sequencing (GBS) (Elshire *et al.* 2011), can generate thousands of markers and help to increase genome saturation. Based on either single marker analysis (SMA) or single-QTL models, such as simple (IM) and composite (CIM) interval mapping, these studies applied univariate methods for single environments or jointly adjusted means across multiple environments. In contrast, multitrait multiple interval mapping (MT-MIM) (Silva et al. 2012) is expected to simultaneously search for multiple QTLs while considering the correlated phenotypic structure of multiple environment trial (MET) data. Such multivariate QTL models are usually more powerful when detecting QTLs and can provide insights on QTL by environment (Q × E) interactions (El-Soda *et al.* 2014), and have been successfully applied in several studies (*e.g.*, Sabadin *et al.*, 2012; Liu *et al.*, 2016).

The present study aimed to assess the genetic architecture of bioenergy traits in a RIL population derived from a cross between two sweet sorghum cultivars. In order to do so, a GBS-based high-density linkage map was constructed, eight bioenergy traits were evaluated in three field trials, and multitrait QTL mapping was carried out. We were able to observe that the presence of Q × E interactions was either due to different size effects across all trials or due to nonsignificant effects in one or two trials. Finally, we investigated putative genes within QTL regions that might further the understanding of the genetic basis of bioenergy traits and facilitate the development of marker-assisted selection.

## Materials and methods

### Plant material and experimental design

A RIL population consisting of 272 individuals was obtained from a cross between the inbred lines Brandes and Wray by single-seed descent (Brim 1966). Both sweet sorghum parents are photoperiod insensitive, *i.e*., flowering occurs regardless of day length, and show high total sugar content. However, they contrast in the quantity and composition of specific sugars. Wray has high sucrose and low reducing sugar content (RSU), while Brandes has low sucrose and high RSU, which is one of the reasons it has traditionally been used for syrup production in North America (Monk *et al.* 1984; Silva *et al.* 2017). These cultivars are also contrasting to aluminum toxicity response, where Brandes is tolerant, and Wray is susceptible (R. E. Schaffert, personal communication).

In three field trials (designated as environment henceforth), 223 RILs plus the two parents were evaluated at F_2:6_ generation, using a 15 × 15 lattice design with three replicates, totaling 675 plots each trial. The plots were composed of two 5-m rows, spaced by 0.70 m. RIL parents were used as checks in all trials, which were conducted in three crop seasons in Sete Lagoas, MG, Brazil. Trial 1 (T1; 2011) and 2 (T2; 2011/2012) were sown on February 3, 2011, and December 13, 2011, respectively, in the same experimental area (geographic coordinates –19.449760, –44.176479). Trial 3 (T3; 2013/2014) was sown on October 17, 2013, in a different experimental area (geographic coordinates −19.473939, −44.174442). Climatic data related to each trial (Supplementary Figure S1) have shown that T1 had shorter photoperiod, lower temperatures, and more irregular rainfall when compared to the other trials. From soil analysis data from the two experimental areas (Supplementary Table S2), we noticed that T1 and T2 were in acid subsoil with high levels of aluminum toxicity, whereas T3 was in alluvial soil with no subsoil acidity and aluminum toxicity. Sowing was done in a no-tillage system and plants received supplemental irrigation throughout the cycle. Before sowing, 400 kg . ha^−1^ of fertilizer 8-28-16 (NPK) were applied to the soil, and 200 kg . ha^−1^ of surface-applied urea fertilizer was applied 20 days after sowing.

The evaluated traits were days to flowering (FLW), plant height (HGH) in cm, fresh biomass yield (FBY) in t · ha^−1^, juice extraction yield (JUC) in %, total soluble solids (BRX) in °Brix, sucrose content (SUC) in % of juice, RSC (RSU) in % of juice, and fibers (FIB) in %. FLW was the number of days after sowing to 50% of plants of the whole plot with 50% of panicle shedding pollen. HGH was taken at harvest and consisted of the average of the whole plot. FBY was measured in kilograms per plot and converted into tons per hectare (t · ha^−1^). JUC extraction was carried out in a hydraulic press with a minimum and constant pressure of 250 kgf · cm^−2^ for 1 minute, using 0.5 kg of fresh biomass from eight plants without panicles sampled randomly per plot. BRX, FIB, SUC, and RSU were obtained from the JUC extraction (*i.e*., eight pooled plants per plot). BRX was measured in a digital refractometer (°Brix). FIB was estimated by Tanimoto’s method (1964). In T1, SUC and RSU were evaluated, respectively, in a polarimeter after clarification of the juice with an aluminum-based mixture, and distillation with Fehling A and B, according to Lane and Eynon’s method (1934). The results were used to develop a calibration curve for a near-infrared spectroscopy (NIRS) method, using a Büchi NIRFlex N-500 FT-NIR spectrometer (Flawil, Switzerland), equipped with a diffuse reflectance accessory. Subsequent trials (T2 and T3) were analyzed using the validated model for SUC and RSU via NIRS, adapted from Guimarães *et al.* (2014, 2016).

### Phenotypic analyses

Mixed model phenotypic analyses were performed separately for each trial. Parameter estimates were obtained via the restricted maximum-likelihood method using GenStat software (v16.1) (VSN International 2014), considering the following model for each trait:

$$y_{\mathrm{ijk}}=\mu +r_{k}+b_{j\left(k\right)}+t_{i}+s_{i\left(jk\right)}+\varepsilon_{\mathrm{ijk}}$$

where $y_{\mathrm{ijk}}$ is the observed phenotypic value for individual i in block j and replicate k; μ is the overall mean; $r_{k}$ is the fixed effect of the kth replicate (k=1,…, K; K=3); $b_{j\left(k\right)}$ is the random effect of the jth block (j=1,…, J; J=15) at replicate k, with $b_{j\left(k\right)}∼N(0,\sigma_{b}^{2})$; $t_{i}$ is the fixed effect of the ith individual (i=1,…,n; n=225); $s_{i\left(jk\right)}$ is the fixed effect of the covariate number of plants of genotype i in block j at replicate k; and $\varepsilon_{\mathrm{ijk}}$ is the residual random effect, with $\varepsilon_{\mathrm{ijk}}∼N(0,\;\sigma^{2})$. Residual plots were visually inspected in order to evaluate model assumptions, especially normal distribution and the presence of outliers. Genotype adjusted means were estimated via best linear unbiased prediction (BLUP) and used for QTL mapping, as described later.

In order to compute heritability values, we separate the $t_{i}$ effect in the model above into the random effects $g_{i}$ of the RIL genotypes ($i=1,\ldots ,n_{g}$; $n_{g}=223$), with $g_{i}∼N(0,\sigma_{g}^{2})$, and the fixed effects $c_{i}$ of parental genotypes ($i=n_{g}+1,\ldots ,\;n_{g}+n_{c}$; $n_{c}=2$). Block ($\sigma_{b}^{2}$), genetic ($\sigma_{g}^{2}$), and residual ($\sigma^{2}$) variance components were then estimated from this model. The coefficient of variation (CV) and the generalized heritability ($h^{2}$) (Cullis *et al.* 2006) were computed for each trial following the respective equations:

$$CV=\frac{\sqrt{\sigma^{2}}}{\bar{y}}\times 100\;\;\;\;\;\;\;\;\;\;\;\;\;\;\;\;\;\;\;\;h^{2}=1-\frac{\bar{v}B\mathrm{LUP}}{2\sigma_{g}^{2}}$$

where $\overset{-}{y}$ is the phenotypic mean, and $\overset{-}{v}B\mathrm{LUP}$ is the average variance of the difference between two BLUPs.

The investigation of G × E interactions for each trait involved the study of genetic covariances across trials. We extended the single-trial model above (with genotypes as random) to a multitrial model:

$$y_{\mathrm{ijkl}}=\mu +h_{l}+r_{k(l)}+b_{j\left(kl\right)}+c_{i}+hc_{il}+g_{il}+s_{i\left(\mathrm{jkl}\right)}+\varepsilon_{\mathrm{ijkl}}$$

where $h_{l}$ is the fixed effect of the lth trial (l=1,…,L; L=3) and all the other design effects were nested within trials, except for $g_{il}$, which represents both the main genotype effect and the interaction term between genotypes and trials. Again, checks ($c_{i}$) and check-by-trial interactions ($ch_{il}$) were treated as fixed effects, whereas RIL genotypes within trials were treated as random effects, with $g∼N(0,G_{L}⊗I_{n_{g}})$, where $g=[g_{11},\ldots ,g_{n_{g}L}]\prime$ and $G_{L}$ is the L×L matrix of genetic variance–covariances between genotype performances in different trials. The $G_{L}$ matrix can be assumed as having identical or heterogeneous variances and covariances (Supplementary Table S3), which provides indications for G × E interactions. Matrix structures were selected for each trait by looking for the smallest values of Akaike (AIC) (Akaike 1974) and Bayesian (BIC) (Schwarz 1978) information criteria.

Additionally, in order to examine the pattern of genotypic mean responses across trials, the BLUEs from each trial were evaluated based on the genotype plus genotype-by-environment (GGE) interaction biplot method (Yan and Kang 2002) using the R package GGEBiplotGUI v1.0-9 (Frutos *et al.* 2014). This method based on single value decomposition allows studying genotype-by-environment (G × E) interactions from the correlation between variables given by the angles between their vectors in a biplot. Positively correlated traits will show vectors with angles between 0 and 90°, while negatively correlated traits will show vectors with angles greater than 90°. No correlation is observed when vectors form angles equal to 90° (orthogonal). Therefore, changes in vector orientations for the same trait across trials indicate G × E.

### GBS and linkage map construction

GBS was performed on the HiSeq™ 2000 platform (Illumina, Inc.) by the Genomic Diversity Facility of Cornell University (Ithaca NY, USA) according to the protocol described by Elshire *et al.* (2011). The *Ape*KI restriction enzyme was used for the construction of genomic DNA libraries including 272 RILs and three replicates of each parent. Single-nucleotide polymorphisms (SNPs) calling was performed using the Tassel-GBS pipeline (Glaubitz *et al.* 2014), implemented in the Tassel software v4.3.8 (Bradbury *et al.* 2007). For this, 64-bp tags were aligned against the sorghum reference genome v2.1 (Paterson *et al.* 2009) and SNPs were recorded in HapMap files, resulting in a total of 461,241 markers (Supplementary Table S4). Heterozygous genotypes were set to missing.

We ran QTL analyses (as described next) based on genotype probabilities from imputed and nonimputed markers, with the latter reconciled into a genetic map. On the one hand, the computation of genotype probabilities from imputed markers relies on each single marker information alone. On the other hand, a genetic map takes the marker dependencies into account, using a multipoint approach based on a hidden Markov model (Lander and Green, 1987; Jiang and Zeng, 1997). In the first analysis, missing data from the HapMap files were imputed using the NPUTE software (Roberts *et al.* 2007). We tested imputation window sizes ranging from 5 to 150 markers, and those showing the highest imputation accuracy were selected. The selected imputation window sizes ranged from 12 (chromosome 7) to 18 (chromosome 4) markers, with an average accuracy of 98.4%. The imputed markers were filtered at a 40% minor allele frequency and redundant markers were also excluded, resulting in 66,007 markers, ranging from 2,790 (chromosome 8) to 9,489 (chromosome 2) (Supplementary Table S4).

In the second analysis, a genetic map was estimated using the R package Onemap v2.1.2 (Margarido *et al.* 2007). From the raw HapMap files (nonimputed markers), we filtered out those markers with more than 25% missing data, noninformative parents (either missing or inconsistent with population genotypes), redundant information, or high segregation distortion (α=0.05, Bonferroni corrected). Pairwise recombination fractions were calculated among the remaining markers and used to group them considering recombination fraction < 0.35 and log of the odds (LOD) score > 8. Markers in disagreement with the chromosome origin of the majority of linked markers were considered false positives and filtered out. The final multi-point estimation was performed according to the marker order from each chromosome. A hidden Markov model probability error of 1% was adopted in order to accommodate GBS-related errors as similarly done by Bilton *et al.* (2018).

### QTL mapping and gene search

The genotype adjusted means for each trial were used separately in univariate MIM analyses (Kao *et al.* 1999) and jointly in multivariate MT-MIM analyses (Silva et al. 2012). While MIM models would map QTLs for each independent trial, MT-MIM models allow for the detection of QTLs based on the information of all trials simultaneously. In order to distinguish between MIM and MT-MIM results, we added the respective suffixes to QTL names for trial-specific (T1, T2, or T3) and multiple-trial (MT) analyses. QTLs were numbered sequentially within the trait-analysis combination according to the genome order (*e.g*., FLW-T1.1 and FLW-MT.1 are the first QTLs for FLW from T1-MIM and MT-MIM analyses, respectively).

QTL identification was based on the following model as implemented in an under-development R package called OneQTL (Silva et al. 2012):

$$y_{ei}=\mu_{e}+\sum_{r=1}^{R}\left(a_{er}x_{\mathrm{air}}\right)+\varepsilon_{ei}$$

where $y_{ei}$ is the adjusted mean for individual i (i=1, 2,…, I; I=223) in the environment e (e=1,…,E; E=3 for MT-MIM or E=1 for MIM); $\mu_{e}$ is the intercept for each environment *e*; $a_{er}$ is the additive genetic effect of the QTL r (r=1, 2, …, R) in the environment e; $x_{\mathrm{air}}$ is an indicator variable for the additive genetic effect a of QTL r in individual i, and assumes values –1 and 1 for genotypes referring to parents Wray and Brandes, respectively; and $\varepsilon_{i}∼N(0,\Sigma_{E})$ is the residual error, where $\Sigma_{E}$ is the variance-covariance matrix of E environments. The Haley–Knott Regression method (Haley and Knott 1992) was used for model fitting and the maximum-likelihood method to estimate the parameters.

Multiple QTL models were built based on a forward–backward procedure, testing the significance of a putative QTL effect based on the conditional probabilities estimated either at each imputed marker from the physical map or at every centiMorgan (cM) interval along the genetic map. We used two window sizes of 100 and 200 kb in the analyses based on the physical map, while a window size of 10 cM was adopted in the analyses based on the genetic map. These window sizes comprise high linkage disequilibrium (LD) regions neighboring QTLs already in the model. Genome-wide significance levels of 5% and 1% were considered for the forward-selection and backward-elimination procedures, respectively, based on the score statistics (Zou and Zeng 2008).

For genetic map-based analyses, ∼95% support intervals were obtained as the region around the highest LOD score (QTL peak) with a drop of 1.5, *i.e*., LOD-1.5 (Silva et al. 2012). In multivariate models, nonsignificant QTL effects were removed based on the seemingly unrelated regression coefficient method (Zellner 1962). In other words, if a QTL had a significant effect on a specific environment, then only this environment-specific QTL effect was kept in the model. LOD score values of MIM and MT-MIM final models and comparison of QTL effects among traits were plotted using the R package ggplot2 (Wickham 2016).

Using the genetic map-based MT-MIM results, we searched for candidate genes within a 10-kbp window on both sides of each QTL peak on the sorghum genome using PhytoMine tool by Phytozome (https://phytozome.jgi.doe.gov/phytomine) (Accessed: 2021 May 31). In order to focus our search into bioenergy-related traits, we also looked into 150 differentially expressed genes (DEGs) in three tissues (top 50 genes each) of sweet sorghum and 50 known sucrose-related genes identified as such by Cooper *et al.* (2019), to see whether they were within our QTL support intervals. As our SNPs were mapped against a previous sorghum genome (v2.1), we converted their coordinates to the current release (v3.1.1) using the CrossMap software (Zhao *et al.* 2014) as implemented in the Assembly Converter (http://ensembl.gramene.org/Sorghum_bicolor/Tools/AssemblyConverter) (Accessed: 2021 May 31) by Gramene (Tello-Ruiz *et al.* 2018).

## Results

### Phenotypic analyses and G × E interactions

**Figure 1:**
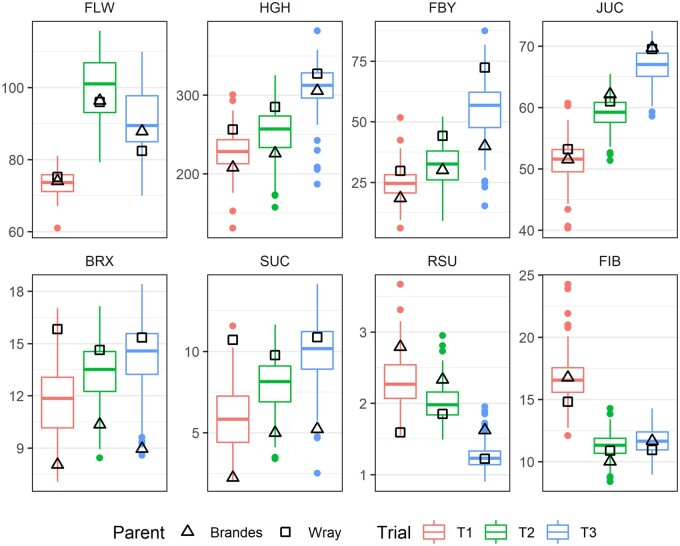
Boxplots of adjusted means of Brandes × Wray sweet sorghum RILs evaluated in three trials (T1, T2, T3). Traits: days to flowering (FLW), plant height (HGH, in cm), fresh biomass yield (FBY, in t · ha^−1^), juice extraction yield (JUC, in %), total soluble solids (BRX, in °Brix), sucrose (SUC, in %), reducing sugar content (RSU, in %) and fibers (FIB, in %).

Heritability for FLW, HGH, BRX, and SUC ranged from moderate to high ($h^{2}>0.74$), while relatively lower heritability was consistently observed within trials for FBY, JUC, RSU, and FIB ($h^{2}<0.68$) (Table 1). Coefficients of variation (CV) were found to be as low as 2.07 for FLW-T1 and as high as 25.70 for FBY-T1. Transgressive segregation was detected in the mapping population for all traits (Figure 1). BRX and SUC were the most phenotypically contrasting traits among the parents, resulting in a broad variation among individuals across trials. Great parental phenotypic divergence was also observed for FBY-T3 and RSU-T1. On the other hand, FLW, JUC, and FIB did not present such marked contrast between the parents, even though great variability for FLW was observed among RILs for T2 and T3. In fact, FLW was similar for both parents in T1 (74 days) and T2 (96 days), with shorter photoperiods, but slightly different in T3 (88 and 82 days for Brandes and Wray, respectively), with longer photoperiod (Supplementary Figure S1). T3 exhibited the highest variation for FLW and consistently resulted in higher adjusted means for HGH, FBY, JUC, BRX, and SUC (Figure 1). T1 had more unfavorable environmental conditions overall (Supplementary Figure S1), namely lower temperature and no rainfall on the second half of that season, which added up to shorter photoperiod to explain lower yield-related traits, such as HGH and FBY.

**Table 1:** Genetic ($\sigma_{g}^{2}$) and residual ($\sigma^{2}$) variance components, coefficient of variation (CV), generalized heritability ($h^{2}$) for Brandes × Wray sweet sorghum recombinant inbred lines evaluated in three trials (T1, T2, and T3)

Traits	T1				T2				T3			
	$\sigma_{g}^{2}$	$\sigma^{2}$	CV	$h^{2}$	$\sigma_{g}^{2}$	$\sigma^{2}$	CV	$h^{2}$	$\sigma_{g}^{2}$	$\sigma^{2}$	CV	$h^{2}$
FLW	8.55	2.31	2.07	0.89	60.24	56.48	7.57	0.76	68.32	8.17	3.15	0.93
HGH	476.10	168.80	5.70	0.88	790.10	381.10	7.74	0.85	577.30	222.60	4.81	0.84
FBY	21.73	39.69	25.70	0.60	48.37	53.84	22.92	0.68	77.30	147.00	21.91	0.56
JUC	7.76	10.37	6.31	0.64	3.44	5.03	3.79	0.63	5.43	5.15	3.40	0.65
BRX	3.40	2.18	12.67	0.79	2.38	2.11	10.88	0.76	3.05	1.68	9.02	0.82
SUC	3.02	2.07	24.50	0.79	2.22	2.22	18.70	0.74	3.09	1.69	12.91	0.82
RSU	0.08	0.16	17.46	0.55	0.01	0.14	18.76	0.18	0.02	0.03	13.20	0.65
FIB	1.90	2.26	9.02	0.68	0.54	0.95	8.63	0.54	0.63	0.60	6.62	0.73

Traits: days to flowering (FLW), plant height (HGH, in cm), fresh biomass yield (FBY, in t · ha^−1^), juice extraction yield (JUC, in %), total soluble solids (BRX, in °Brix), sucrose (SUC, in %), reducing sugar content (RSU, in %), and fibers (FIB, in %).

Regarding the selection of genetic variance–covariance matrix $G_{L}$, we have found evidence toward structures with heterogeneous variances across trials for all traits expect SUC (Supplementary Table S3). In addition, heterogeneous covariances between trials were also found in the selected matrix structures for five traits (FLW, FBY, JUC, RSU, and FIB), whereas the structures with homogeneous covariances were selected for the three remaining traits (HGH, BRX, and SUC). Despite the need for the estimation of additional parameters, the selection of more complex structures when compared to the simplest, naïve assumption of identical homogeneous genetic variance across trials and no covariance between them is an indication that G × E interactions were relevant for all traits.

G × E interactions were also assessed using GGE biplot (Supplementary Figure S2). Adjusted means from different trials were more positively correlated for FLW, HGH, FBY, and RSU in general, indicating low G × E interaction. Implying greater G × E interaction, the adjusted means for JUC and FIB revealed smaller positive correlations, especially from T1 in respect to T2 and T3, and for BRX and SUC, especially from T3 in respect to T1 and T2. Together with Pearson correlations (Supplementary Figure S3), the GGE biplot indicated three groups of highly positively correlated traits: JUC and RSU (forth quadrant), FLW, HGH, and FBY (third quadrant), and BRX and SUC (second quadrant). FIB from T1 and T2 (first quadrant) exhibited a high negative correlation with the traits on the third quadrant, while FIB-T3 was negatively correlated with those on the forth quadrant. In addition, traits on the forth quadrant, which includes RSU, showed a negative correlation with those on the second quadrant, which contains SUC and BRX.

### Physical map *vs* genetic map-based QTL analyses

MT-MIM physical map-based analyses resulted in 52 and 51 QTLs when adopting the respective mapping window sizes of 100 and 200 kb. All chromosomes harbored at least one QTL, except chromosomes 2 and 7. Chromosomes 1, 3, 6, and 9 harbored most QTLs (40 and 37 when using the respective 100 and 200-kb windows). Six regions (on chromosome 3 for SUC and RSU, on chromosome 6 for FLW, and on chromosome 9 for JUC, BRX, and SUC) appeared to have two closely linked QTLs under 100-kbp window (Supplementary Figure S4), which were resolved into only one QTL under 200-kb window (Supplementary Figure S5), as a result of a better false positive control. LOD scores greater than 10 were observed in 21 and 22 QTLs for the respective 100 and 200-kb windows, with the highest for FLW on chromosome 6 (LOD=50.41). However, such high LOD scores are very unlikely to appear in our dataset, with a sample size of 223, and were regarded as statistical artifacts. Flat peaks were observed on chromosomes 3 (BRX, SUC, and RSU), 6 (FLW, HGH, FBY, BRX, SUC, and FIB), and 10 (FLW, HGH, FBY, and JUC), making it harder to clearly define QTL peaks (Supplementary Figures S4 and S5). These chromosomes have shown broad regions of high LD as measured by squared correlation coefficients ($r^{2}$) (Supplementary Figure S6).

**Figure 2:**
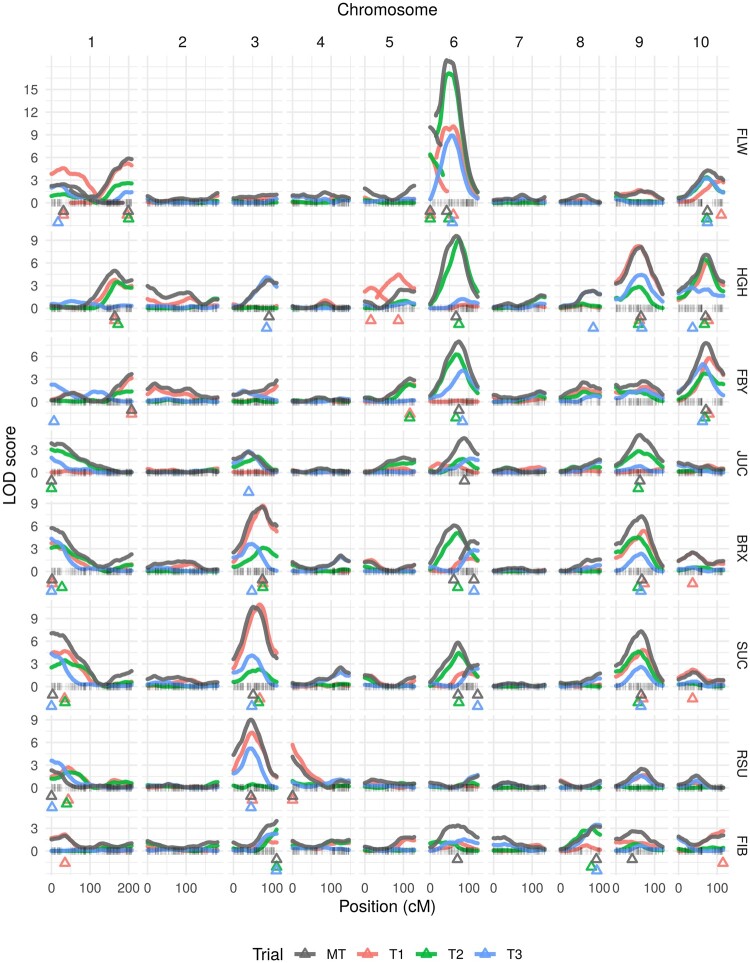
Genetic map-based LOD score profiles of multiple interval mapping (MIM) for individual (T1, T2, T3) and multiple trials (MT) for Brandes × Wray sweet sorghum RILs. Triangles represent mapped QTLs. Traits: days to flowering (FLW), plant height (HGH, in cm), fresh biomass yield (FBY, in t · ha^−1^), juice extraction yield (JUC, in %), total soluble solids (BRX, in °Brix), sucrose (SUC, in %), reducing sugar content (RSU, in %), and fibers (FIB, in %).

In order to construct a genetic map from nonimputed HapMap files, 97.5% of markers were filtered out, mostly due to the high rate of GBS-derived missing data. Out of the 11,417 remaining markers, 4,194 were nonredundant and, out of these, 3,876 did not show segregation distortion. The final map consisted of 3767 markers, spanning 1368.83 cM, with a density of 2.75 SNPs per cM (Supplementary Files S4 and S5). The number of markers per chromosome ranged from 212 (chromosome 8) to 594 (chromosome 1), which were also the shortest (102.28 cM) and the longest (207.67 cM) groups, respectively (Supplementary Table S4). Using such a genetic map, MT-MIM analyses detected a total of 33 QTLs (Table 2 and Figure 2). Five chromosomes (1, 3, 6, 9, and 10) harbored 31 QTLs for several traits, with additional QTLs on other two chromosomes, 4 (RSU) and 8 (FIB). LOD scores ranged from 2.35 (for RSU on chromosome 1) to 18.74 (for FLW on chromosome 6). The QTLs with the highest LOD scores were located on chromosome 3 for BRX (8.56), SUC (10.54) and RSU (9.02), chromosome 6 for FLW (18.87 and 10.00), HGH (9.57), and FBY (7.99), and chromosome 9 for HGH (8.09).

**Table 2:** MT-MIM for Brandes × Wray sweet sorghum recombinant inbred lines evaluated in three trials (T1, T2, and T3)

Trait	QTL	Chr	Position (cM)	Marker (bp)	LOD	T1		T2		T3	
						Effect	PVE	Effect	PVE	Effect	PVE
FLW	1	1	30.00	11,093,514	2.44	–1.69	2.80	sur	sur	sur	sur
	2	1	198.00	71,594,007	5.85	2.72	8.95	5.27	3.79	4.63	3.31
	3	6	0.00	382,696	9.89	–4.81	25.46	–14.05	24.46	–10.96	16.86
	4	6	42.65	4,049,408	18.74	6.83	32.45	27.69	60.03	22.73	45.89
	5	10	74.91	53,882,121	4.29	–2.04	2.72	–7.90	4.58	–8.09	5.45
HGH	1	1	162.83	64,727,343	4.98	17.99	4.91	21.66	3.73	sur	sur
	2	3	92.14	69,453,408	3.75	sur	sur	sur	sur	–21.52	4.51
	3	6	67.00	44,949,491	9.57	sur	sur	38.38	9.16	sur	sur
	4	9	64.70	49,204,605	8.09	–32.69	12.03	–25.59	3.86	–31.07	7.77
	5	10	70.00	48,678,282	7.08	–25.17	7.48	–40.70	10.25	–22.77	4.38
FBY	1	1	206.69	73,162,697	3.67	4.39	6.23	3.97	2.79	sur	sur
	2	6	73.91	46,121,015	7.97	sur	sur	10.88	10.12	12.05	6.14
	3	10	71.00	48,678,282	7.77	–7.58	8.98	–8.66	6.40	–14.49	8.87
JUC	1	1	0.00	1,581,214	3.87	sur	sur	1.54	5.76	1.32	3.07
	2	6	88.00	48,478,843	4.53	sur	sur	–1.91	4.39	–2.76	6.59
	3	9	62.40	48,103,357	4.98	sur	sur	2.76	7.75	2.35	4.03
BRX	1	1	0.00	1,581,214	5.78	–1.30	5.38	–1.09	4.98	–1.31	6.24
	2	3	75.14	65,279,548	8.54	–3.14	12.94	–1.70	4.97	–1.49	3.31
	3	6	61.00	42,731,400	6.02	sur	sur	2.18	8.83	sur	sur
	4	6	113.18	52,779,223	4.00	0.96	2.01	sur	sur	1.46	5.30
	5	9	67.00	49,406,423	7.30	–2.62	9.11	–1.99	6.90	–1.67	4.20
SUC	1	1	1.71	2,709,413	7.10	–1.49	7.83	–1.05	4.75	–1.43	7.09
	2	3	51.00	58,433,131	10.52	–3.27	15.25	–1.37	3.27	–2.23	7.00
	3	6	71.64	45,898,926	5.78	1.25	2.45	2.10	8.45	sur	sur
	4	6	122.80	54,328,118	2.87	sur	sur	sur	sur	1.22	4.09
	5	9	65.26	49,406,423	7.29	–2.42	8.66	–2.05	7.58	–1.72	4.35
RSU	1	1	0.00	1,553,727	2.35	sur	sur	sur	sur	0.10	4.20
	2	3	45.22	57,882,869	9.02	0.52	10.71	sur	sur	0.21	8.63
	3	4	0.00	273,007	4.13	0.29	6.73	sur	sur	sur	sur
FIB	1	3	112.04	73,490,974	3.98	1.12	3.62	0.75	5.17	0.77	5.54
	2	6	70.04	45,740,799	3.38	sur	sur	sur	sur	0.90	5.16
	3	8	92.62	52,308,546	3.28	sur	sur	0.49	2.98	0.62	4.82
	4	9	42.00	6,770,591	2.62	1.49	4.53	sur	sur	sur	sur

Chr: chromosome; LOD: log of the odds; PVE: proportion of the phenotypic variance explained by the QTL, in %; sur: seemingly unrelated regression coefficients (Zellner 1962). Traits: days to flowering (FLW), plant height (HGH, in cm), fresh biomass yield (FBY, in t · ha^−1^), juice extraction yield (JUC, in %), total soluble solids (BRX, in °Brix), sucrose (SUC, in %), reducing sugar content (RSU, in %), and fibers (FIB, in %). Positive effects represent alleles inherited from Brandes and negative effects represent alleles from Wray.

Support intervals could not be computed from physical map-based analyses, as LOD scores from individual marker tests are not continuous along the chromosomes, neither LOD-1.5 seemed appropriate due to the high LOD scores. Despite these statistical artifacts, several physical map-based QTL were included within QTL regions from genetic map-based analyses (Supplementary File S9). It is worth mentioning that LD has been effectively considered when reconciling linked markers into a genetic map (Supplementary Figure S6), leading to a better false positive control and statistical sounding LOD scores. Therefore, large physical regions without clear peaks for QTLs from physical map-based analyses (Supplementary Figures S4 and S5) have been resolved in the context of genetic map-based analyses (Figure 2).

### Genetic architecture of bioenergy traits

**Figure 3:**
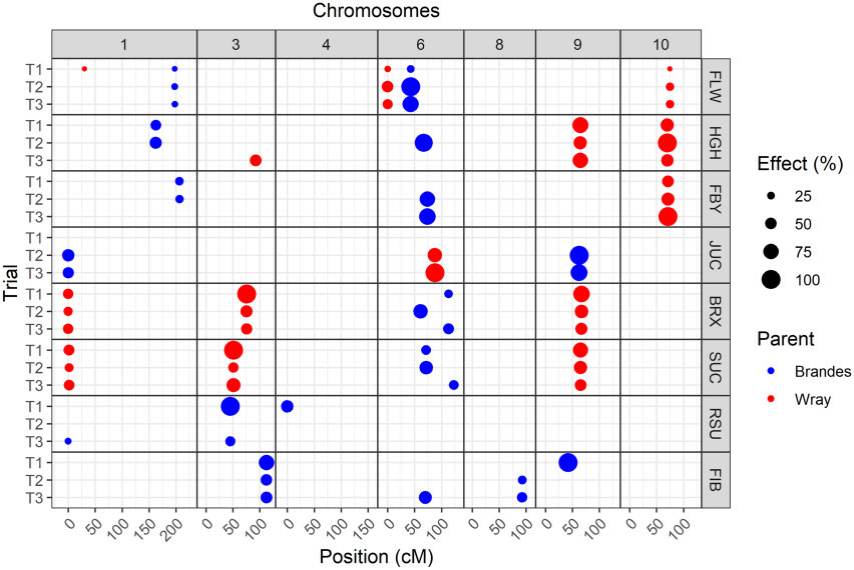
MT-MIM for Brandes × Wray sweet sorghum recombinant inbred lines evaluated in three trials (T1, T2, T3). Dots represent QTL peaks positioned along the genetic map (in cM), where size denotes the QTL effects relative to the largest within trait (in %) and color denotes the parental origin of favorable alleles. Traits: days to flowering (FLW), plant height (HGH, in cm), fresh biomass yield (FBY, in t · ha^−1^), juice extraction yield (JUC, in %), total soluble solids (BRX, in °Brix), sucrose (SUC, in %), reducing sugar content (RSU, in %), and fibers (FIB, in %).

From genetic map-based analyses, we detected five QTLs for FLW: two on chromosome 1, two on chromosome 6, and one on chromosome 10. The two QTLs on chromosome 6 explained together most of the trait variation from each trial (57.91%, 84.49%, and 62.75%, respectively), with favorable alleles coming from different parents each (FLW-MT.3 from Wray and FLW-MT.4 from Brandes). Although effects for all trials were significant for four QTLs, their magnitude differed across trials, with smaller effects in T1 (Figure 3). For example, for FLW-MT.4, Brandes allele contributed with 6.8 days in T1, but with 27.7 and 22.7 days in T2 and T3, respectively. FLW-MT.1 showed a significant effect on T1 alone, which was colocated not only with FLW-T1.1, but also with FLW-T3.1 from MIM analyses.

For HGH, one QTL was found for each chromosome 1, 3, 6, 9, and 10. HGH-MT.4 and HGH-MT.5 were found to be significant in all three trials, both with favorable alleles coming from the taller parent, Wray. Sill contributing with height, Wray allele had a significant effect on T3 for HGH-MT.2. With Brandes allele contributions, HGH-MT.1 influenced T1 and T2, and HGH-MT.3 only affected T2. Interestingly, for the same trial T2, HGH-MT.3 contributed with 38.4 cm, which was comparable to the HGH-MT.5 effect with 40.7 cm. These results were corroborated by MIM analyses, where QTLs specific to the corresponding trial were identified (*i.e*., HGH-T1.1 and HGH-T2.1 colocated with HGH-MT.1, HGH-T3.1 colocated with HGH-MT.2, and HGH-T2.2 colocated with HGH-MT.3), with no statistical evidence for QTL in the other trial(s).

Regarding the three QTLs for FBY, on chromosomes 1, 6, and 10, FBY-MT.1 and FBY-MT.2 have shown nonsignificant effects for T3 and T1, respectively, whereas only FBY-MT.3 has influenced all trials. For FBY-MT.1 and FBY-MT.2, Brandes allele effects were comparable across trials, whereas for FBY-MT.3, Wray allele effect was more pronounced on T3. On the other hand, all three QTLs for JUC, on chromosomes 1, 6, and 9, did not show a significant effect for T1. In fact, only two of them (JUC-MT.1 and JUC-MT.3) colocated with MIM results (JUC-T2.1 and JUC-T2.2). Brandes alleles contributed with JUC-MT.1 and JUC-MT.3, whereas Wray alleles contributed with JUC-MT.2. In addition to these three QTL, physical map-based analyses for JUC have revealed additional putative QTLs on chromosomes 3, 5, and 10, that were completely missed in the genetic map-based analyses.

BRX and SUC had five QTLs each: one on each chromosome 1, 3, and 9, and two on chromosome 6. Corresponding support intervals from all five QTL overlapped when comparing both traits (Supplementary Figure S7). BRX/SUC-MT.1, BRX/SUC-MT.2, and BRX/SUC-MT.5 had significant effects for all trials, which were all contributed by Wray (higher in sucrose). T1 was more impacted by BRX/SUC-MT.2 in comparison with the other two trials. Brandes alleles for SUC/BRX-MT.3 and SUC/BRX-MT.4 influenced trials differently, as SUC-MT.3 had effects on T1 and T2 only, but the latter was the only one influenced by BRX-MT.3. Similarly, SUC-MT.4 had a single effect on T3, while BRX-MT.4 had significant effects on both T1 and T3. For this specific chromosome 6, MIM only detected an exclusive QTL for T2 (BRX/SUC-T2.3) and T3 (BRX/SUC-T3.3). Physical map-based analyses revealed additional QTL on chromosomes 1 and 3 for both traits, and on chromosome 9 for BRX.

For QTLs from both RSU and FIB, there was an exclusive contribution from Brandes, which is the parent with higher reducing sugar and fiber contents. For RSU, there were three QTLs, one on each chromosome 1, 3, and 4. No QTL had a significant effect on T2, as RSU-MT.1 and RSU-MT.3 only affected T3 and T1, respectively, and RSU-MT.3 has shown a greater effect on T1 than on T3. For FIB, out of four QTLs, on chromosomes 3, 6, 8, and 9, only one, FIB-MT.1, was identified with consistent effects for all trials. For the remaining three QTLs, FIB-MT.3 has shown significant effects on both T2 and T3, whereas FIB-MT.2 and FIB-MT.4 have impacted only T3 and T1, respectively. These two QTLs were not, in fact, detected during MIM analyses for any specific trial. From the physical map-based analyses, an additional QTL was declared on chromosome 3 for RSU.

### Gene search

Our gene search within a 10 kb-window neighboring the QTL peaks returned 500 genes (Supplementary File S10). In addition to 42 transcription and translation factors associated with our QTLs, we found at least 26 homologs predicted to encode enzymes involved in carbohydrate pathways which can be targeted in further validation studies. Furthermore, two lists from Cooper *et al.* (2019) guided us when looking for additional genes. From a list of 150 DEGs in sweet sorghum (Supplementary File S11), most of QTL support interval overlaps were with transcription factors (at least 44), while only two were with sugar-related genes: UDP-glucosyl transferase (Sobic.001G030600, colocated with several QTL on chromosome 1 proximal), and sugar transporter (Sobic.003G213000, colocated with RSU-MT.2). From a list of 50 known sugar-related genes (Supplementary File S12), 18 of them were found to be positioned within the support intervals of 25 of our QTLs. Sugar transporters are putative products of 10 of these genes (*e.g*., Sobic.003G213000, colocated with RSU-MT.2, Sobic.003G269300, colocated with HGH-MT.2, BRX-MT.2, and SUC-MT.2, Sobic.008G193300, colocated with FIB-MT.3, and Sobic.009G143500, colocated with several QTLs on chromosome 9). The remaining eight genes encode enzymes participating in carbohydrate pathways. In addition to other glucosyl transferases (Sobic.001G084500, colocated with on chromosome 1, and Sobic.010G205100, colocated with HGH-MT.5 and FBY-MT.3) and glycosyl hydrolases (Sobic.001G099700, colocated with several QTL on chromosome 1, and Sobic.004G004800, colocated with RSU-MT.3), we also found homologs for glucose-6-phosphate isomerase (Sobic.001G071800, colocated with around QTLs on chromosome 1) and sucrose synthase (*e.g*., Sobic.001G378300, colocated with HGH-MT.1).

## Discussion

The MT-MIM approach used here provides a proper way of studying QTL stability across environments (Silva et al. 2012; Liu *et al.* 2016). In our study, 14 out of 33 QTLs had significant effects in all trials, where three exhibited the same parental origin of the favorable alleles from Brandes (positive signs) and 11 from Wray (negative signs) (Figure 3). The remaining 19 QTLs have shown nonsignificant QTL effects in one or two specific trials. The significant effects had the same sign across trials for all QTLs (noncrossover interactions), where Brandes and Wray provided superior alleles for 16 and 3 QTLs, respectively. Both parents contributed favorable alleles for almost all evaluated traits except RSU and FIB, whose contributing alleles were inherited from Brandes, the parent known by its relatively high RSU. Brandes provided more favorable alleles for FBY, JUC, RSU, and FIB, whereas Wray provided most of the favorable alleles for FLW, HGH, BRX, and SUC. Interestingly, these four traits exhibited the most significant QTLs, which corresponded to greater effects and PVEs (Table 2).

The existence of QTLs with consistent major effects across all trials has shown a tendency to exhibit smaller G × E interaction (El-Soda *et al.* 2014). Proportionally, FLW, BRX, and SUC presented more QTLs with significant effects across all trials, followed by HGH, FBY, and FIB, but there were no QTLs for JUC and RSU with significant effects for all trials (Table 2). We investigated the extension of G × E interactions based on both covariances (through $G_{L}$ selection) and mean ranks (via GGE biplots) genetic differences across trials. From the multitrial mixed model analyses, we noticed that there was no evidence for the simplest matrix structure of identical variances and no covariances between trials for any of the traits (Supplementary Table S3). Thus, some degree of G × E interactions was observed for all traits, although simpler variance–covariance structures were selected for HGH, BRX, and SUC, whereas more complex structures were selected for FLW, FBY, JUC, RSU, and FIB. From GGE biplot analysis (Supplementary Figure S2), we observed that G × E interaction was lower for FLW, HGH, FBY, and RSU in comparison to the remaining traits. FLW, HGH, and FBY presented major QTLs contributing significantly to the phenotypic variation. Greater G × E interaction was observed for BRX and SUC, which possibly contributed to the detection of a couple of QTLs with no effects in some trials. Finally, more pronounced G × E interaction was detected when comparing trial-specific means for FIB and JUC, for which most or all QTLs were considered nonsignificant for at least one trial.

A major effort by Mace *et al.* (2019) has compiled QTL results from previous studies for several traits in sorghum in a consensus map and integrated it with genomic information in a database called Sorghum QTL Atlas (https://aussorgm.org.au/sorghum-qtl-atlas/) (Accessed: 2021 May 31). From the Atlas, we learned that a total of 122, 100, and 26 QTLs colocated with our QTLs for FLW, HGH, and the remaining bioenergy-related traits, respectively (Supplementary File S13). These observations confirmed QTL hotspots on chromosomes 1, 6, and 10 with 49, 140, and 36 QTLs reported to date. In addition to chromosomes 3 and 9, these five chromosomes harbored most QTLs detected in the present study (Figure 2 and Table 2). QTLs may cluster together due to reduced recombination events, likely related to selection processes during domestication, as already reported for chromosome 6 (Hamblin *et al.* 2011; Bouchet *et al.* 2017). In fact, some *Maturity* (*Ma*) (Quinby 1974; Rooney and Aydin 1999) and *Dwarf* (*Dw*) (Quinby 1974) loci might be linked, such as *Ma1* (FLW-MT.4) and *Dw2* (HGH-MT.3) on chromosome 6 (Ritter *et al.*, 2008; Murphy *et al.*, 2011; Bai *et al.*, 2017) leading to phenotypically correlated traits. The vegetative growth stops at flowering, which explains how late flowering would enable taller plants. Similarly, taller plants would generally correspond to an increase in biomass, so that correlation between HGH and FBY might also be due to sharing colocated QTLs (Figure 3 and Supplementary Figure S3). Later flowering is also believed to be related to greater sugar accumulation (Burks *et al.* 2015; Mocoeur *et al.* 2015), but just a few common minor QTL regions, namely on chromosomes 1 and 6 (Figure 2), seemed to underly both FLW and SUC/BRX. There have been maturity loci reported on chromosomes 3 and 9 (Mace *et al.* 2013; Bouchet *et al.* 2017), where major SUC/BRX QTLs lie, but they were likely monomorphic in our population.

Regarding the QTL regions previously identified for SUC and BRX, those on chromosome 6 shared the same genomic locations with BRX/SUC-MT.3 (Ritter *et al.*, 2008; Bai *et al.*, 2017) and BRX/SUC-MT.4 (Shiringani *et al.* 2010), in addition to BRX/SUC-MT.2 on chromosome 3 (Murray *et al.* 2008b; Felderhoff *et al.* 2012; Harris-Shultz *et al.* 2015). On the other hand, BRX/SUC-MT.1 and BRX/SUC-MT.5 can be regarded as novel QTLs as their support intervals did not overlap previously identified QTL regions on chromosomes 1 (Ritter *et al.* 2008; Shiringani *et al.* 2010; Guan *et al.* 2011; Felderhoff *et al.* 2012) and 9 (Shiringani *et al.* 2010; Felderhoff *et al.* 2012). For JUC, the QTLs on chromosomes 1 (Guan *et al.* 2011) and 6 (Mace and Jordan, 2011; Burks et al., 2015; Mocoeur *et al.*, 2015) had been described before, while the one on chromosome 9 was newly found. For RSU and FIB, only related traits were used to investigate previous QTL occurrences. For glucose content (a reducing sugar), two QTLs previously reported were found to colocate with our QTLs for RSU on chromosomes 3 (Shiringani *et al.* 2010) and 4 (Wang *et al.* 2013b). Moreover, two QTLs for acid detergent fiber colocalized with the ones for FIB on chromosomes 6 and 9 (Shiringani and Friedt 2011), and one QTL for neutral detergent fiber colocated with the one on chromosome 8 (Murray *et al.* 2008a). Murray *et al.* (2008a) also identified a QTL on chromosome 3 for cellulose (a structural carbohydrate), but it did not colocalize with our newly discovered FIB-MT.3. These authors suggested that the loci controlling the expression of fiber content have a pleiotropic effect on sugar concentration in the stem, which would explain the correlation between BRX/SUC and FIB.

To perform a more thorough search for candidate genes, Murray *et al.* (2009) have designed markers based on more than a hundred enzymes associated with starch and sucrose metabolism, including sugar transporters (Kanehisa 2006). The only significant association they found was for BRX on chromosome 1, approximately 12 kb away from a glucose-6-phosphate isomerase homolog (Sobic.001G071800), which overlaps with the support interval of several QTLs in our study, including BRX/SUC-MT.1 and RSU-MT.1. From lists of DEGs (Supplementary File S11) and known sugar-related genes (Supplementary File S12) in sweet sorghum (Cooper *et al.* 2019), we also found UDP-glycosyl transferase genes (Sobic.001G084500 and Sobic.001G030600) in the same region, in addition to other homologs within our QTL regions on chromosomes 1, 3, 9, and 10. UDP-glucose is directly involved in the synthesis of sucrose, using fructose-6-P to generate sucrose-P by the action of sucrose phosphate synthase (SPS). Subsequently, sucrose-P is converted into sucrose by the enzyme sucrose phosphate phosphatase. Sucrose can also be converted to fructose and UDP-glucose to be used in cellular processes, such as respiration and polymer biosynthesis, including cell wall components (Wang *et al.* 2013a).

We also observed putative genes in the same genomic regions of other QTLs for some additional traits (Supplementary File S12). RSU represents the total of glucose and fructose, generated from sucrose by the action of glycosyl hydrolases family 32 (invertases), whose putative genes have been mapped close to QTLs on chromosome 1 (Sobic.001G099700), and colocalized with QTLs from BRX/SUC, on chromosome 4 (Sobic.004G004800). Another glycosyl hydrolase homolog (Sobic.006G160700) has been found associated with BRX-MT.4 on chromosome 6. In this same chromosome, Brenton *et al.* (2016) described other two glycosyl hydrolases family 5 (Sobic.006G122200 and Sobic.006G122300, at ∼49.7 Mb) with SNPs associated with nonfibrous carbohydrates. Similarly, two independent studies (Xia *et al.* 2018; Zhang *et al.* 2018) described a NAC transcriptional factor (Sobic.006G147400, at ∼51.8 Mb) as the candidate gene underlying the *Dry* (*D*) gene in sorghum. These genes lie within JUC-MT.2, BRX/SUC-MT.4, and FIB-MT.2 support intervals (Supplementary File S8) and show how this QTL hotspot can affect stem composition. FIB represents the structural biomass (as cellulose, hemicelluloses, and lignin found in cell walls), in which not only cellulose synthases (such as Sobic.009G063400 associated with FIB-MT.4) might participate, but also bidirectional sugar transporters. These transporters, such as SWEET10, are involved in the export of sucrose across the plasma membrane to the apoplasm (cell wall space) (Chen *et al.* 2012). SWEET10-like genes were found within QTL support intervals for FIB on chromosomes 3, 8, and 9 (Sobic.003G377700, Sobic.008G193300, and Sobic.009G143500), with the latter colocalized with QTLs from BRX/SUC.

Our results not only validated some of the previously identified QTLs, but also allowed the discovery of novel regions underlying the variation of bioenergy-related traits. Newly discovered QTLs might be a result of both mapping methodology (multivariate analysis) and population genetic background (exclusive sweet sorghum parents). Moreover, based on the GGE biplot analysis, it was possible to observe the existence of G × E interaction across the evaluated trials, which highlights the importance of using an appropriate QTL mapping strategy for multiple environments. The QTLs associated to sugar composition and content in the juice consistently found across trials have great potential use in selection strategies in sweet sorghum breeding programs focusing on bioenergy purposes, especially since Wray and Brandes are sweet sorghum lines known worldwide. Knowledge on QTL stability will leverage breeding programs’ decisions toward broadly adapted cultivars as more diverse environments are investigated. The putative associated genes, highlighted in this work, will also allow for further studies aiming to better understand the genetic control of bioenergy traits, and may provide the basis for marker-assisted selection for these traits in sweet sorghum.

## Data availability

Supplementary Files S1 and S2 contain imputed and nonimputed HapMap files used for the physical map and genetic map-based analyses, respectively. Supplementary File S3 contains phenotypic adjusted means. Supplementary File S4 contains map information. Supplementary File S5 contains the heatmaps of each respective linkage group. Supplementary Files S6, S7, and S8 provide detailed information on MIM and MT-MIM results for each physical map (100- and 200-kb window sizes) and genetic map (10-cM window size) based analyses, respectively. Supplementary File S9 contains colocated QTLs across MIM and MT-MIM based on physical and genetic map analyses. Supplementary File S10 contains the list of putative genes within a 10-kb window on each side of QTLs from genetic map-based analyses. Supplementary Files S11 and S12 contain lists of DEGs and sucrose-related genes (Cooper *et al.* 2019), respectively, along with colocated QTLs from our study. Supplementary File S13 contains the list of previously reported QTLs according to the Sorghum QTL Atlas (Mace *et al.* 2019) along with colocated QTLs from our study. Supplemental material is available at figshare: https://doi.org/10.25387/g3.15183750.

## Funding

Funding for this study was provided by the Brazilian Agricultural Research Corporation (Embrapa, Brazil) and SWEETFUEL project (Sweet Sorghum: An alternative energy crop), supported by the European Commission in the 7th Framework Programme (GA #227422). V.F.S. has received a PhD scholarship from the Brazilian Coordination for the Improvement of Higher Education Personnel Foundation (CAPES). G.S.P. has received a PhD scholarship from São Paulo Research Foundation (FAPESP) award #2012/25236-4, and a research scholarship from the CAPES Foundation award #88887.369781/2019-00. A.A.F.G. was awarded a fellowship of research productivity granted by the National Council for Scientific and Technological Development (CNPq), Brazil.

## Author contributions

J.V.M., R.E.S., and C.M.B.D. conceived and designed the study. V.F.S., R.A.C.P., M.L.F.S., B.A.B., and R.W.N. collected data. V.F.S., G.S.P., M.M.P., L.D.C.E.S., A.A.F.G., and C.M.B.D. analyzed data. V.F.S. and G.S.P. drafted the manuscript. M.M.P., J.V.M., A.A.F.G., and C.M.B.D. critically reviewed the manuscript. All authors read and approved the manuscript.

## Conflicts of interest

The authors declare that there is no conflict of interest.
